# Coordinate Autophagy and mTOR Pathway Inhibition Enhances Cell Death in Melanoma

**DOI:** 10.1371/journal.pone.0055096

**Published:** 2013-01-30

**Authors:** Xiaoqi Xie, Eileen P. White, Janice M. Mehnert

**Affiliations:** 1 The Cancer Institute of New Jersey, New Brunswick, New Jersey, United States of America; 2 Division of Medical Oncology, Department of Medicine, University of Medicine and Dentistry of New Jersey-Robert Wood Johnson Medical School, Piscataway, New Jersey, United States of America; 3 Department of Molecular Biology and Biochemistry, Rutgers University, Piscataway, New Jersey, United States of America; 4 University of Medicine and Dentistry of New Jersey, Robert Wood Johnson Medical School, Piscataway, New Jersey, United States of America; Istituto Nazionale per le Malattie Infettive, Italy

## Abstract

The phosphatidylinositol 3-kinase/AKT/mammalian target of rapamycin (PI3K/AKT/mTOR) pathway promotes melanoma tumor growth and survival while suppressing autophagy, a catabolic process through which cells collect and recycle cellular components to sustain energy homeostasis in starvation. Conversely, inhibitors of the PI3K/AKT/mTOR pathway, in particular the mTOR inhibitor temsirolimus (CCI-779), induce autophagy, which can promote tumor survival and thus, these agents potentially limit their own efficacy. We hypothesized that inhibition of autophagy in combination with mTOR inhibition would block this tumor survival mechanism and hence improve the cytotoxicity of mTOR inhibitors in melanoma. Here we found that melanoma cell lines of multiple genotypes exhibit high basal levels of autophagy. Knockdown of expression of the essential autophagy gene product ATG7 resulted in cell death, indicating that survival of melanoma cells is autophagy-dependent. We also found that the lysosomotropic agent and autophagy inhibitor hydroxychloroquine (HCQ) synergizes with CCI-779 and led to melanoma cell death via apoptosis. Combination treatment with CCI-779 and HCQ suppressed melanoma growth and induced cell death both in 3-dimensional (3D) spheroid cultures and in tumor xenografts. These data suggest that coordinate inhibition of the mTOR and autophagy pathways promotes apoptosis and could be a new therapeutic paradigm for the treatment of melanoma.

## Introduction

Melanoma is a very aggressive tumor with notoriously poor prognosis once disease becomes metastatic [1]. Despite recent advances in the treatment of melanoma, available therapies result in responses that are not durable, with median progression-free survival (PFS) on the order of 5.5 months [2], or that are ineffective in a majority of patients [3]. This has necessitated the identification and incorporation of novel pathways and new approaches to enhance the activity of targeted therapies.

The PI3K/AKT/mTOR signaling pathway is a central pathway promoting cell growth, motility, protein synthesis, survival, and metabolism in response to hormones, growth factors and nutrients. PI3K activates the serine/threonine kinase AKT, which in turn through a cascade of regulators results in the phosphorylation and activation of the serine/threonine kinase mTOR. mTOR, in turn, controls a diverse array of effector pathways that promote cell growth [4], [5]. The PI3K/AKT/mTOR pathway is dysregulated in many types of cancer, including melanoma, and is associated with poor prognosis [6], [7], [8], [9], [10], [11], [12]. Pharmacologic inhibition of PI3K/AKT/mTOR pathway components thus becomes an attractive approach for melanoma treatment. Among agents that interfere with PI3K/AKT/mTOR signaling, inhibitors of mTOR are furthest in clinical development and have demonstrated efficacy in renal cell carcinomas as well as in patients with neuroendocrine tumors of pancreatic origin and in postmenopausal patients with hormone receptor positive breast cancer [13] [14], [15], [16]. CCI-779, an analogue of rapamycin, was approved by the Food and Drug Administration for treatment of renal cancer with poor prognostic features when a survival benefit was seen compared with interferon [14]. Despite the active state of the PI3K/mTOR pathway, to date, studies of CCI-779 in melanoma have not shown promise [17], [18], indicating that discovery and exploitation of novel survival pathways and mechanisms of resistance would be necessary for further successful development of this agent.

Autophagy is induced by multiple anticancer agents [19], [20], especially mTOR inhibitors [21], [22], as a tumor survival-promoting mechanism. When autophagy is induced by agents that block signaling pathways such as the PI3K pathway that mimic starvation, recycling of intracellular components by autophagy can promote survival [23]. As such, autophagy is a potential resistance mechanism that may be abrogated to increase the cytotoxicity of mTOR inhibition. Through autophagy, cellular components including proteins and organelles such as mitochondria are sequestered in double membrane bound autophagosomes and delivered to lysosomes for degradation and recycling [24]. This catabolic cellular “self eating” process removes cellular waste and provides substrates to sustain energy homeostasis and building blocks for biomass generation [25]. Autophagy is a necessary mammalian survival mechanism, highlighted by the failure of mice deficient in the essential autophagy gene *atg5* to survive the neonatal starvation period [26]. Under normal conditions, autophagy is active at low levels to remove the occasional damaged organelle or unfolded protein to prevent their toxic accumulation [27], [28]. Under stressful conditions such as nutrient deprivation, hypoxia, or other sources of cellular stress such as chemotherapy or targeted therapies, autophagy is dramatically induced as a protective mechanism to maintain homeostasis and viability [29]. Autophagy is also induced in insufficiently vascularized tumors, localized preferentially to hypoxic tumor regions, where deficiency in autophagy compromises tumor cell survival [30], [31], [32]. Thus, tumor cells activate and utilize autophagy to survive in the stressed tumor microenvironment [30].

The mTOR complex has been known as a key regulator of autophagy for more than a decade. mTOR is a sensor of nutrient levels to promote growth when nutrients are present, and to block autophagy by interacting with and inhibiting the ULK1 complex [25]. Pharmacologic inhibition of mTOR can thus induce changes in cells similar to starvation or that recapitulate aspects of hypoxia. mTOR inhibition promotes dissociation of mTOR from the complex of ATG13 with ULK1 and ULK2. This frees ULK1/2 to activate FIP200, a protein crucial for autophagosome formation, and initiate autophagy [33], [34], [35], [36]. It thus follows that tumor cells that have been “primed” to undergo autophagy by mTOR inhibition would be more likely to undergo increased cell death when autophagy is impaired.

Flux through the autophagy pathway can be blocked by administration of the antimalarial lysosomotropic agent hydroxycholoroquine (HCQ), which inhibits the final step of autophagy, the fusion of autophagosomes with lysosomes [25], [37]. HCQ treatment thereby leads to the accumulation of autophagosomes that may accelerate tumor cell death. This approach has been the subject of multiple investigations [38], [39], [40], [41], [42], [43], [44], [45] [46]which indicate that addition of the antimalarial choloroquine (CQ) promotes tumor cell death in preclinical models. Clinical benefit of the addition of CQ to anticancer therapy was suggested in a phase III study of glioblastoma patients treated with radiation and carmustine with and without this agent, where the median overall survival was 24 months in the CQ treated group, versus 11 months in the placebo treated patients [47]. This trial was not adequately powered to detect differences in overall survival, but its results taken together with the emerging preclinical data underscore the importance of further investigation of this therapeutic strategy [20].

Many tumor types have high basal levels of autophagy [38], [46], including melanoma, in which a high autophagic index has been associated with tumor aggressiveness and poor outcome [48], [49]. Certain tumors may be more reliant on autophagy and as such, more sensitive to coordinate mTOR and autophagy inhibition. Given these observations we sought to investigate the effects of coordinate mTOR and autophagy inhibition in preclinical models of melanoma.

## Results

### Human Melanoma Cell Lines and Melanoma Patient Specimens Show High Levels of Autophagosomes

**Figure 1:**
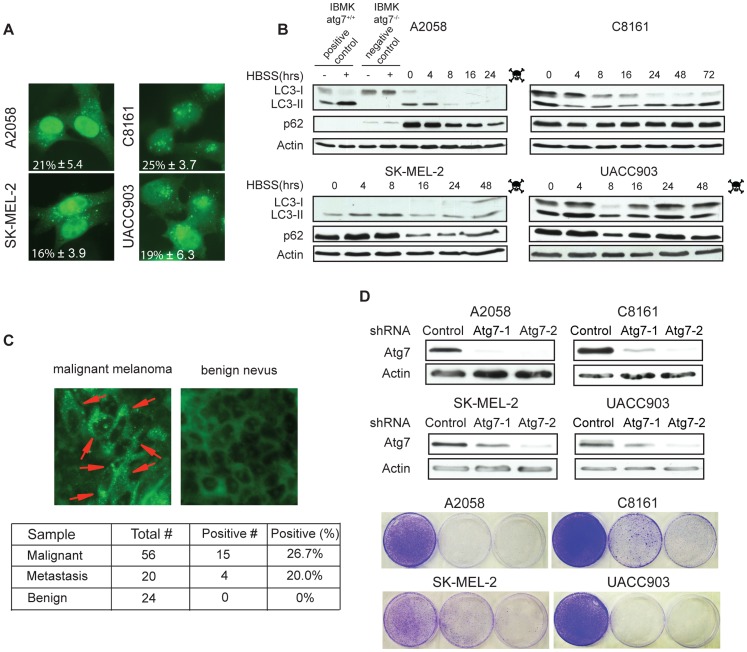
Human melanoma cell lines and melanoma patient specimens showed high levels of basal autophagy. (A) Melanoma cell lines were transiently transfected with an EGFP-LC3 expressing plasmid and grown in normal medium. 24 hours after transfection, the cells were fixed and the autophagosomes in the cells were visualized by the presence of LC3 puncta under fluorescence microscopy, with the percentages of cells showing LC3 puncta (mean ± SD) indicated. All tested cell lines showed positive punctation indicating high basal autophagy. (B) Western blot shows that melanoma cell lines have high basal LC3-II and p62 protein levels. Upon HBSS induced starvation, increased cleavage of LC3-I to LC3-II indicative of autophagy induction and decreased or unchanged p62 levels were seen. Extracts from autophagy competent and autophagy deficient iBMK cells served as positive and negative controls. (C) Immunofluorescence staining for endogenous LC3 on a human melanoma tissue microarray. Punctate LC3 localization for autophagosomes is indicated by red arrows (top panel). The percentages of specimens with punctate LC3 staining in malignant, metastatic and benign nevus groups are indicated in the table (bottom panel). (D) Western blot showed decreased expression levels of Atg7 (top panel) and impaired clonogenic survival (bottom panel) in response to lentiviral shRNA knockdown of the essential autophagy regulator Atg7.

To determine if basal levels of autophagy are elevated in melanoma, the human melanoma cell lines C8161 (wild type *Braf^V600E^* and *N-Ras^Q61R^*), A2058 (*Braf^V600E^* mutation, PTEN deleted), UACC903 (*Braf^V600E^* mutation, PTEN deleted) and SK-MEL-2 (*N-Ras^Q61R^* mutation), representative of a range of melanoma genotypes, were transfected with the autophagy reporter enhanced green fluorescent protein (EGFP)-LC3 plasmid. Cells were then grown in normal medium, fixed, and examined by epifluorescence microscopy for the presence of autophagosomes visualized by the processing and autophagosome membrane translocation of fluorescent LC3 [50]. Upon autophagy induction, LC3-I is proteolytically cleaved and lipidated to form LC3-II, which localizes to the autophagosome membrane and is a measure of increased autophagosome number that can be visualized by the appearance of punctate structures. All four cell lines, regardless of genotype, showed high basal levels of autophagasome puncta, with the percentages of cells showing LC3 puncta (mean ± SD) ranging from 16–25% (Fig. 1A), and high ratios of detectable LC3-II/LC3-I bands on Western blot (Fig. 1B). These data are comparable to the high levels of basal autophagy in other human cell lines shown in our previous work [46].

To assess the potential for autophagy induction under conditions of stress and to measure flux through the pathway, growth medium from melanoma cell lines was replaced with Hanks’ Balanced Salt Solution (HBSS) to induce autophagy. Cells were then collected at different time points and assessed by Western blot to detect LC3-I conversion to LC3-II and p62 levels. p62 is an autophagy substrate degraded by autophagy, with levels inversely correlating with autophagic activity [27]. p62 binds polyubiquinated proteins (cargo), aggregating them by oligomerizing, and it also binds to LC3 on the autophagosome membrane, to target aggregates containing cargo to autophagosomes for degradation. Immortal baby mouse kidney (iBMK) cells wild type or deficient for the essential autophagy gene *atg7* were used as positive and negative controls, respectively [27], [46]. All melanoma cell lines tested showed increased autophagy induction when treated with HBSS as demonstrated by conversion of LC3-I to LC3-II that was not seen in autophagy deficient cells, with slight variation in the timing of this event (Fig. 1B). A2058, SK-MEL-2 and UACC903 cells showed increased LC3-II levels 4 hours after starvation that was initially sustained, which then subsequently decreased as cell death occurred between 24 and 48 hours. In contrast, C8161 cells showed increased LC3-II/LC3-I ratio at 8 hours post starvation, with cells remaining alive up to 72 hours. p62 protein levels decreased in A2058, SK-MEL-2 and UACC903 cell lines during starvation, whereas in C8161 cells, the p62 protein level stayed unchanged. Reduction in p62 levels can correlate with autophagy induction, but may not always if other modes of p62 regulation come into play. These experiments suggest that melanomas may have high basal autophagy, with further induction of autophagy observed under metabolic stress.

To discern if high basal levels of autophagy could also occur in human melanoma samples, human melanoma tissue microarrays with 100 cores were stained by immunofluorescence for LC3 as described previously [51] (Fig. 1C). Autophagosomes were present in 20% of metastatic and 26.7% of primary melanoma lesions that showed high levels of autophagosomes by positive LC3 punctate staining. In contrast, no positive LC3 punctate staining was observed in the benign nevi (Fig. 1C). This indicates that autophagosomes are not commonly found in benign nevi and that human melanomas exhibit possible evidence of upregulation of basal autophagy in a subset of samples. These high levels of autophagsosomes can indicate high levels of autophagy, or alternatively a block to autophagic flux that is not possible to distinguish in specimens of fixed tumor tissue.

### Knockdown of Atg7 Impairs Melanoma Cell Growth

To investigate the role of autophagy in melanoma cell growth and survival, we knocked down expression of the essential autophagy gene *atg7* by infecting melanoma cell lines with two different shRNA-expressing lentiviruses directed against *Atg7* or a luciferase control. Western blot confirmed that the levels of Atg7 protein were markedly decreased in cells infected with Atg7 shRNA compared to cells infected with luciferase shRNA (Fig. 1D). A clonogenic growth assay showed that cell growth was impaired in parallel with reduced expression levels of Atg7 protein in all tested cell lines (Fig 1D). Similar results were obtained with knockdown of another essential autophagy gene, Atg5 (Supplementary Fig. S1). These data demonstrate that autophagy ablation impaired melanoma cell growth and induced cell death.

### HCQ Blocks Autophagic Flux that is Increased by Allosteric mTOR Inhibition

**Figure 2:**
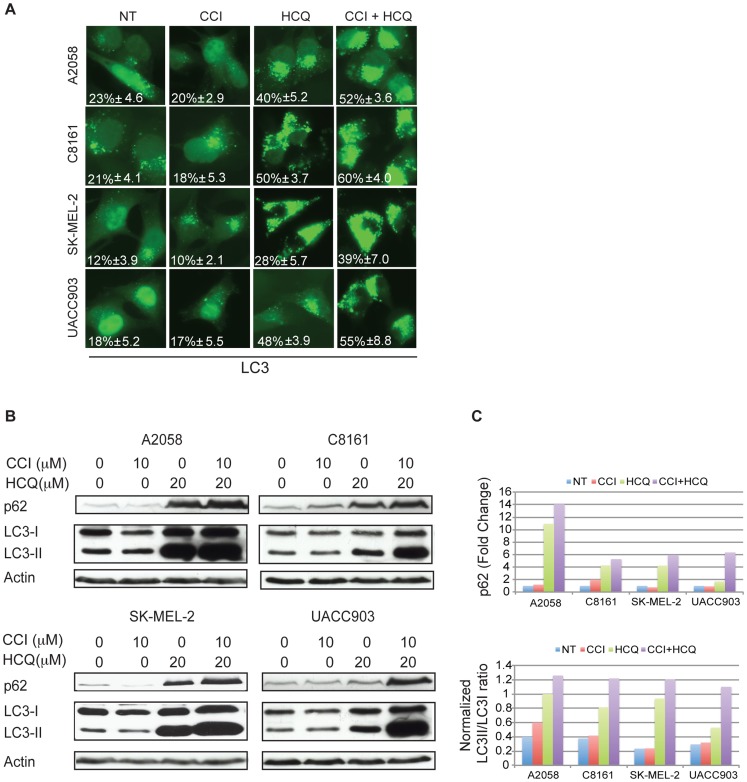
Autophagic flux is induced by CCI-779 and blocked by HCQ. (A) Melanoma cell lines transiently transfected with an EGFP-LC3-expressing plasmid were seeded on coverslips and treated with 10 µM of CCI-779 and 20 µM of HCQ, alone and in combination for 24 hours. Autophagosomes were visualized by the presence of LC3 puncta. The drug combination treatment showed more autophagosome accumulation than either single agent alone. The percentages of cells showing LC3 puncta (mean ± SD) are indicated. (B) Western blot showed that the combination treatment resulted in considerable increases in LC3-II and increases in p62 protein levels compared to treatment with single agent HCQ alone, indicating the blockade of autophagic flux. (C) Quantitation of actin-normalized changes in p62 (upper panel) and the ratio of LC3-II/LC3-I (lower panel) after single and combination treatment in comparison to the untreated control.

To prove that autophagy could be modulated in melanoma cell lines through coordinate autophagy and mTOR inhibition, melanoma cell lines were transfected with EGFP-LC3 and then treated with CCI-779 or HCQ alone or with the combination of both agents (Fig. 2A). No significant changes in autophagosome formation were noted upon treatment with CCI-779 alone. HCQ blocks flux through the autophagy pathway by blocking fusion of autophagosomes with lysosomes, resulting in increased autophagosome numbers that cannot be cleared. As expected, autophagosome accumulation was noted after treatment with HCQ, but combination treatment with both HCQ and CCI-779 resulted in higher numbers of autophagosomes than HCQ alone (Fig. 2A). Western blot of LC3-II and p62 was performed to further quantitate blockade of autophagy. Indeed, addition of HCQ resulted in profound conversion of LC3-I to LC3-II and increased p62 protein levels (Fig. 2B and C). This effect was greater in cells treated with both CCI-779 and HCQ. Therefore, HCQ blocks flux through the autophagy pathway, which is more pronounced in the setting of autophagy induction by CCI-779.

### Inhibitors of mTOR and Autophagy Demonstrate Synergistic Cytotoxicity in Melanoma Cells

**Figure 3:**
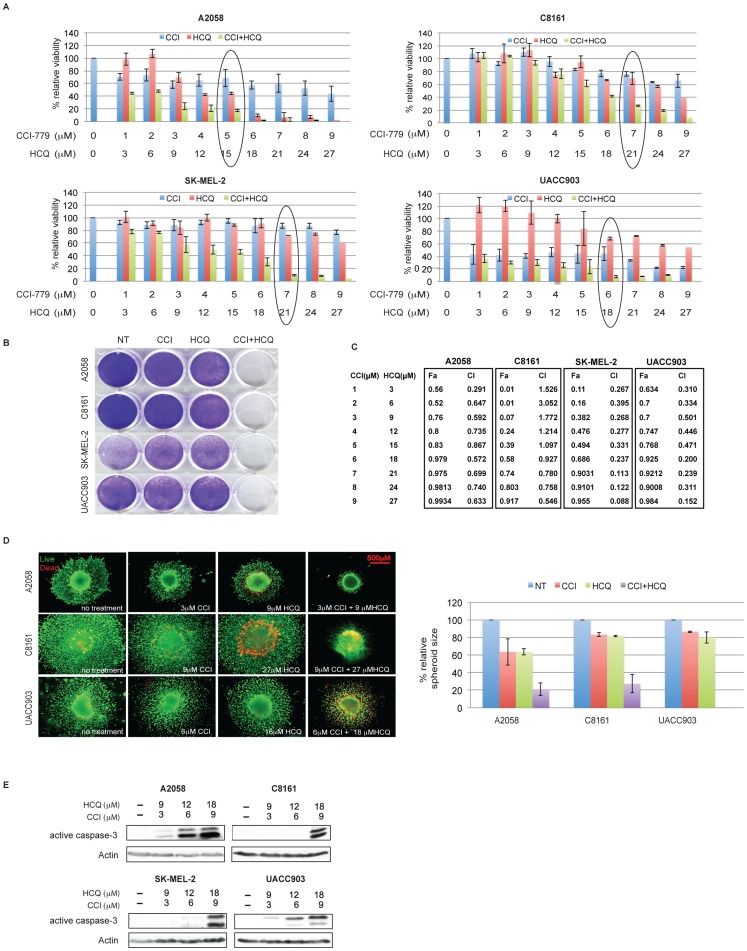
HCQ and CCI-779 synergize to induce melanoma cell death. (A) Melanoma cells (2×10^4^) were plated in 12 well plates, and treated with CCI-779 and HCQ alone and in combination at the concentrations indicated. The cell viability was examined at day 4 by Trypan blue stain based cell counting and normalized to untreated cells at the time zero. Relative viability showed that CCI-779 and HCQ cooperate to promote cell death. (B) Clonogenic survival assay showed that melanoma cells treated with CCI-779 and HCQ in combination at the concentration as circled in (A) impaired colony formation. (C) Synergistic effects were analyzed by CalcuSyn software, with a fixed ratio of CCI- 779 and HCQ concentration (1∶3) as indicated. Experimental points fall mostly below the borderline (CI = 1) indicating strong synergism. (D) Inhibition of melanoma cell growth in three-dimensional culture. Melanoma cell spheroids were grown as described in the Materials and Methods. After 72 hours of incubation with CCI-779 and HCQ alone and in combination, the cells were treated with cell LIVE/DEAD Viability kit wherein living cells stain green and dead cells stain red. Representative 3-D spheroid pictures showed synergism between CCI-779 and HCQ (scale bar = 500 µM) (left), with the graph at right displaying the percentages of spheroid diameters (mean ± SD) normalized to the untreated spheroids. Spheroids of UACC903 disintegrated after the treatment. (E) Combination HCQ and CCI-779 induced cell death via apoptosis. Western blot of melanoma cells treated with HCQ and CCI-779 at indicated concentrations demonstrated increased active caspase-3 indicating apoptotic cell death.

To evaluate the functional consequences of combination treatment with autophagy and mTOR inhibitors, melanoma cells were treated with HCQ and CCI-779 alone and in combination for 72 hours (Fig. 3A). CCI-779 treatment did not increase the dead and viable cells by cell counting, implying that its use predominantly resulted in cytostatic growth arrest rather than cytotoxic cell death, whereas HCQ showed moderate cytotoxic effects. As expected, the co-administration of CCI-779 and HCQ resulted in a dramatic decrease in cell viability that was confirmed with clonogenic assays, with these two drugs cooperating in a dose-dependent manner (Fig. 3A and 3B).

In a fixed concentration ratio (1∶3) between CCI-779 and HCQ, relative viability was analyzed by CalcuSyn software, which performs drug dose-effect (Fa) calculation for determination of Combination Index (CI) (Fig. 3C). CI values of <1,  = 1, and >1 are indicative of synergism, additive, and antagonistic effects, respectively. HCQ and CCI-779 demonstrate synergistic effects in all the cell lines tested, but in C8161 cells, the synergism occurred at relatively higher doses of both agents compared to other cell lines (Fig. 3C). Synergy was observed *in vitro* across a range of in vitro doses of HCQ including doses reported to be clinically achievable, although higher micromolar concentrations of HCQ are possibly inconsistently achievable in humans [49], [52], [53].

As cells grown in three-dimensional (3D) culture resemble *in vivo* conditions more closely than do cells in conventional 2D cultures, melanoma cells were grown in 3D cultures as spheroids and treated with CCI-779 or HCQ alone or with the combination of agents (Fig. 3D). After 72 hours, cell death was imaged by a two-color fluorescence cell-viability assay, with green representing live, and red representing dead, cells. Except for SK-MEL-2 cells that did not form spheroids, the other melanoma cell lines showed little growth inhibition upon treatment of CCI-779 or HCQ alone. However, spheroid cultures treated with the combination of CCI-779 and HCQ showed similar evidence of synergistic viability loss, especially the spheroids from the UACC903 cell line that disintegrated after treatment (Fig. 3D). Thus, HCQ synergizes with CCI-779 to induce cell death.

To determine the mechanism by which CCI-779 and HCQ combination treatment induced cell death, we examined the apoptosis marker cleaved active caspase-3 by Western blot. Treatment of A2058 and UACC903 cells at the 6 and 8 hour time points, respectively, caused a dose-dependent increase in cleaved caspase-3, whereas in SK-MEL-2 and C8161 cells, an increase in cleaved caspase-3 occurred at higher doses of each agent and at later time points (24 hours) (Fig. 3E). In addition, treatment with the necrosis inhibitor necrostatin-1 could not rescue the cell death (Supplementary Figure S2) indicating the cell death was not necrotic as seen with CCI-779 and CQ treatment of human renal cancer cell lines [23].

### Combination Autophagy and mTOR Inhibition Suppresses Melanoma Xenograft Growth

**Figure 4:**
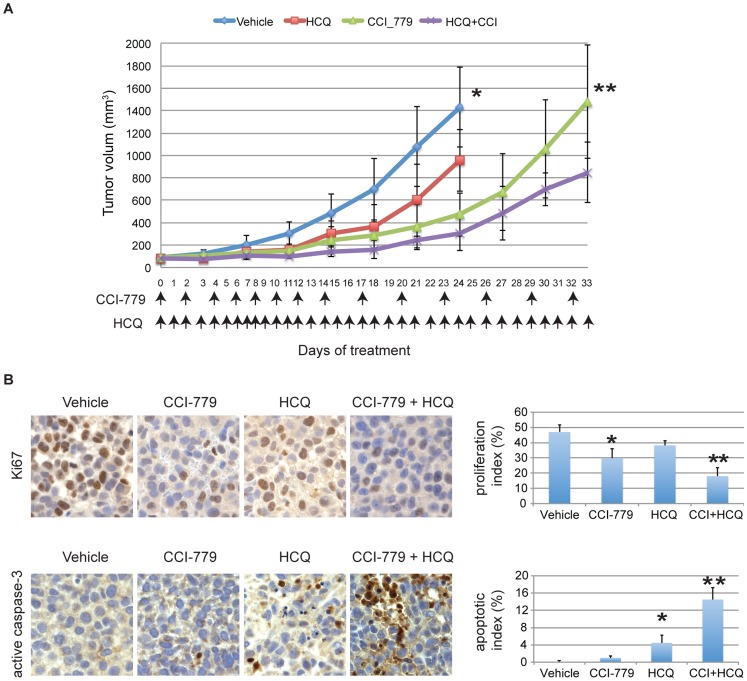
CCI-779 and HCQ in combination inhibit UACC903 tumor xenograft growth. (A) Nude mice bearing 100 mm^3^ tumors were given daily s.c. injection of 65 mg/kg HCQ and 0.5 mg/kg CCI-779 at the days indicated. Tumor size was measured once every three days. Each data point represents the mean ± SE tumor volume from 5 mice, 10 tumors, with tumor suppression greatest in mice treated with both CCI-779 and HCQ (*P = 0.001, **P = 0.014). (B) Representative pictures of immunohistochemical analysis showed that Ki67 was reduced in tumors treated with CCI-779, HCQ either alone or in combination. The proliferation index was calculated as the proportion of Ki67 positive cells among 200 cells in 4 randomly selected fields. Error bars represent standard errors. (*) P = 0.004; (**) P = 0.0013 (t-test) compared with the vehicle control. Active-caspase-3 staining was increased in tumors treated with HCQ, and was further increased in tumors treated with CCI-779 and HCQ. The apoptotic index was calculated as the proportion of active caspase 3 positive cells among 200 cells in 4 randomly selected fields. (*) P = 0.007; (**) P = 0.0014 (t-test) compared with vehicle control.

To investigate the relevance of coordinate autophagy and mTOR inhibition in melanoma tumor growth *in vivo,* a xenograft mouse model of melanoma was treated with CCI-779, HCQ and the combination of agents. Although both A2058 and UACC903 cells were very sensitive to the combination treatment in 2D and 3D cell culture, UACC903 grew less aggressively *in vivo* which allowed us to observe the tumor growth for a longer period of time. UACC903 cells were thus chosen as the representative cell line and were subcutaneously injected into six week old nude mice to monitor growth. When tumors reached around 100 mm^3^, HCQ (65 mg/kg) was injected on a daily basis and CCI-779 (0.5 mg/kg) was administered every other day from days 1–15 and every two days after day 15 as indicated in Fig. 4A, and the kinetics of tumor growth were measured. The HCQ dose selection for the mouse in vivo melanoma (UACC903) tumor growth inhibition was based on the human dose of 200 mg twice daily, a common dose used in clinical practice that can be escalated [53]. HCQ administration suppressed tumor growth compared to the control group (two tailed student’s *t*-test, *P* = 0.001, n = 10). Tumors treated with CCI-779 alone and in combination with HCQ exhibited slower growth compared to the vehicle control group. After day 15, the combination treatment showed significant tumor suppression compared to single agent CCI-779 treatment (two tailed student’s *t*-test, *P* = 0.014, n = 10). No discernible side effects such as weight loss or change in behavior were observed.

To investigate whether the suppression of tumor growth with the combination treatment is also associated with decreased proliferation and increased apoptosis *in vivo* as observed in our *in vitro* models, we stained the tumor sections for Ki67 and active caspase-3 (Fig. 4B, left panel). Treatment with CCI-779 or HCQ either alone or in combination led to a reduction in cell proliferation as demonstrated by decreased Ki67 staining. In addition, both vehicle control and CCI-779-treated tumors showed negligible positive staining for active caspase-3. Tumors from mice treated with HCQ alone showed a modest increase in active caspase-3 that was further increased in tumors from mice treated with the HCQ and CCI-779 combination (Fig. 4B left panel).

To quantitatively compare the proliferation and apoptotic index in vehicle and different treatment groups, the number of Ki67 and caspase-3 positive cells was determined among 200 cells in 4 randomly selected fields (Fig. 4B right panel). In the CCI-779 and HCQ treatment groups, the proliferation indexes decreased. Among them, the CCI-779 and HCQ combination group showed significant differences ((*) P = 0.0040, (**) P = 0.0013). The apoptotic index showed little increase in the CCI-779 group, but was more profoundly increased in HCQ and the HCQ and CCI-779 combination groups. The differences were statistically significant ((*) P = 0.0070, (**) P = 0.0014). Thus, the coordinate inhibition of mTOR and autophagy by treatment with CCI-779 and HCQ reduced tumor volume *in vivo* that was associated with apoptosis.

Taken together, our data demonstrate that CCI-779-induced autophagy contributes to the resistance of melanoma cells to CCI-779 and limits its efficacy. In the absence of the autophagy inhibitor HCQ, CCI-779 caused cytostatic growth arrest and autophagy induction in melanoma cell lines, both of which contributed to the sustained survival of these cells with mTOR inhibition alone. When autophagy was inhibited by HCQ in the presence of CCI-779, autophagosomes accumulated and apoptotic tumor cell death was induced.

## Discussion

Despite recent advances in melanoma treatment, the majority of patients are not cured with current approaches and new options are desperately needed. Although trials of single agent mTOR inhibition produced a 0% stable disease rate, preliminary results in a phase I trial of CCI-779 and HCQ in patients with all solid tumors showed a 74% stable disease rate in melanoma patients at first restaging [54]. These results suggest dual autophagy and mTOR inhibition may be a useful therapeutic paradigm in patients with advanced melanoma.

Autophagy is functional at low levels in normal tissues, but upregulated in settings of metabolic stress, such as the oxygen- and nutrient-deprived microenvironment of tumors. We would expect that in such a microenvironment, autophagy is stimulated and supports tumor growth, whereas autophagy inhibition could suppress tumor growth or lead to tumor death. Therapeutic abrogation of autophagy may thus be most effective in patients whose tumors display high baseline autophagy levels and aggressive behavior. High levels of autophagy are a common feature of multiple tumor types and an independent predictor of poor prognosis in colorectal, pancreatic, and gastrointestinal tumors as well as melanoma [48], [55], [56], [57]. Our data indicate that four of four melanoma cell lines tested, regardless of genotype, showed high basal levels of autophagy and high autophagic flux. Our examination of human melanoma and skin tissue using a 100 core melanoma tissue microarray showed high basal levels of autophagosomes in a subset of melanomas, implying that a subset of human melanomas have the potential for high autophagy levels (Fig. 1C). These results are consistent with other reports, albeit using different techniques, which indicate that the degree of baseline autophagy among human melanomas is heterogeneous [49], [58]. Preclinical models suggest that indeed, aggressive melanomas have higher levels of autophagy and greater response to autophagy inhibition than melanomas which are more indolent [49]. Future studies to develop the means to measure autophagy are needed to investigate this phenomenon in human samples. This represents an active area of current investigation, as the ability to identify precisely which patients might benefit from this therapeutic strategy would be advantageous in the selection of treatment options.

Our results show that genetic inhibition of autophagy through knockdown of the additional essential autophagy components Atg7 and Atg5 impaired melanoma cell growth in multiple cell lines, demonstrating the reliance upon autophagy for survival. Indeed, existing reports of knockdown of the essential autophagy components Atg5 or Atg7 in the literature suggest that this approach results in increased cell death in different melanoma cell lines, although additional metabolic stressors may be required [59], [60], [61]. Knockdown of Atg5 alone was sufficient to induce cell death in aggressive versus indolent melanomas or in pancreatic ductal adenocarcinomas [38], [49], underscoring the possibility that certain tumors may rely more heavily on autophagy for survival than others.

HCQ inhibits autophagy by blocking the fusion of autophagosomes with lysosomes in the final steps of autophagy. This approach is under investigation in clinical trials [19], usually in combination with chemotherapy, radiation therapy or other targeted therapies. We did note decreased viability of cell lines treated with HCQ alone, as well as tumor growth suppression in our xenograft model (Fig. 4A), in keeping with reports of others [38]. However, given the genetic complexity of human tumors and melanomas in particular, it is unlikely this single agent approach would achieve cure. Indeed, our results show that pharmacologic inhibition of autophagy with HCQ dramatically increased the antitumor activity of the mTOR inhibitor CCI-779, creating synergy that converted cytostatic mTOR inhibition to cytotoxicity. This is similar to our previously published findings in renal cell carcinoma models [23] and reflective of reports which indicate that the addition of chloroquine analogs sensitizes tumor cells to anticancer treatments [39], [40], [41], [44], [45], [62], [63].

A major limitation in the translation of coordinate autophagy and mTOR inhibition to the clinic is that the precise mechanism of cell death promotion is unknown. CQ enhances the cytotoxicity of CCI-779 in renal cell carcinoma; this increase in cell death was due to ROS-mediated, RIP kinase-dependent programmed necrosis (necroptosis) [23]. In the studies presented here, administration of CCI-779 and HCQ decreased cell line viability by apoptosis. Similar findings have recently been reported in preclinical models that employed coordinate inhibition of the autophagy and PI3K/AKT/mTOR pathways. The addition of CQ increased PI3K/AKT/mTOR inhibitor-induced cell death in models of malignant peripheral nerve sheath tumors and in mantle cell lymphoma cells that were resistant to Akt/mTOR targeting, resulting in induction of cell death via apoptosis [64], [65]. The newer, dual PI3K/mTOR inhibitors in clinical development are thus of interest given the potential to eliminate feedback activation of AKT which can theoretically allow the cell to escape growth arrest in the presence of mTOR inhibition [20], [44]. Further development of this approach in clinical trials should include the acquisition of paired tissue samples to verify if this same mechanism is operative in human tumors, particularly melanoma, where tissue is often easily procurable for correlative studies that have been the basis of recent progress in the field [66].

Recent advances in the treatment of advanced melanoma have led to the development of a molecular disease model [67] that classifies individual tumors into molecular subtypes which could inform treatment selection. In this backdrop of emerging knowledge, characterizing the role of autophagy as it relates to distinct genetic mutations is essential. Although the small number of cell lines tested precludes definitive conclusions, our data indicate that autophagy can be modulated for therapeutic benefit regardless of tumor genoptype. Indeed, human melanomas reportedly showed no difference in baseline levels of autophagy as detected by number of autophagic vacuoles/cell in patients whose tumors harbored mutations in *BRAF* or *NRAS* or who were wild type for both genes [49], although this study, too, was limited by small numbers, as well as the lack of observed mutations of homozygous deletions of *pten*, the most common mutation in melanoma to impact PI3K/Akt/mTOR signaling [49]. While a recent report suggests that autophagy was less when *BRAF* was mutated in melanoma [59], at the time points that we examined there was significant autophagic flux in melanoma cell lines with mutations in *BRAF* (Fig. 2).

It is possible that autophagy is a process conserved across multiple tumor genotypes, but tumors with more aggressive behavior, such as those with hyperactivation of RAS-MEK signaling, and higher levels of intrinsic metabolic stress are more autophagy-dependent. Studies from our group and others have shown that aggressive cancers such as those with activating mutations in *Ras* have high basal autophagy, suggesting that the genetic events that produce cancer may be inherently stressful [38], [46], [68], [69]. Moreover these tumors can be “addicted” to autophagy for survival [38], [46], as autophagy is necessary to sustain mitochondrial metabolism and energy homeostasis in such tumors. The concept of autophagy dependence may also be applicable to human melanoma cells with RAS-MEK activation driven by mutations in *BRAF ^V600E^*. Sheen et al. showed that when deprived of the essential amino acid leucine, melanoma cells with activated RAS-MEK signaling fail to induce autophagy and trigger caspase-dependent apoptosis instead, a process which was shown to depend on the presence of mutant *BRAF^V600E^*. The survival-promoting role of autophagy was further illustrated by the reactivation of autophagy in oncogenically activated cell lines treated with a MEK or mTOR inhibitor and by a xenograft melanoma model in which the subsequent combination of a leucine free diet and the autophagy inhibitor chloroquine synergistically induced caspase-dependent death [70]. With the majority of melanomas exhibiting RAS-MEK activation through mutations in *N-Ras^Q61R^* or *Braf^V600E^* [66], our findings in conjunction with these studies provide a compelling basis for further exploration of autophagy inhibition in combination with inhibition of RAS-MEK and PI3K/AKT/mTOR signaling, two of the most dysregulated pathways in melanoma. This is particularly relevant as newer, more potent inhibitors of autophagy begin to emerge, such as Lys 05, a dimeric form of chloroquine [52]. In conclusion, further preclinical and clinical investigation of coordinate autophagy and PI3K/AKT/mTOR inhibition as a rational approach to improve therapeutic outcomes in advanced melanoma is warranted.

## Materials and Methods

### Ethics Statement

Animal work was performed using Institutional Animal Care and Use approved protocols, I10-064-8 “Use of Mice for Tumorigenicity”, which was approved by IACUC committee members at the University of Medicine and Dentistry of New Jersey (UMDNJ). Mice were monitored for overall health in response to drug treatment and all efforts were made to minimize suffering. Mice were sacrificed when the tumor exceeded 1500 mm^3^.

### Cell Culture

A2058 and SK-MEL-2 melanoma cell lines were originally purchased from ATCC. C8161 and UACC903 melanoma cell lines were obtained as a gift from the laboratory of James Goydos, M.D., The Cancer Institute of New Jersey. A2058 and C8161 human melanoma cells were cultured in DMEM with 10% FBS and 1% Pen-Strep; UACC903 was cultured in RPMI and SK-MEL-2 in MEM Alpha with 10% FBS and 1% Pen-Strep. All cells were cultured and treated at 37°C in a humidified incubator containing 5% CO_2_. For starvation, cells were washed twice with phosphate buffer saline (PBS, GIBCO/Invitrogen) and placed in HBSS buffer (GIBCO/Invitrogen).

### Antibodies and Reagents

The following antibodies were used in Western blotting and immunohistochemistry: LC3 (Novus Biologicals); p62 [46]; Ki67 (Abcam) activated caspase-3 (Cell Signaling); Atg7 and actin (Sigma-Aldrich); Hydroxychloroquine was purchased from Acros Organics; CCI-779, CCCP, N-acetyl-L-cysteine (NAC) were purchased from Sigma-Aldrich.

### Cell Viability and Clonogenic Assay

Melanoma cells (2×10^4^) were plated in 12 well plates and treated with different doses of HCQ and CCI-779, alone and in combination for 4 days. The viable cell number was determined by a Trypan blue exclusion-based cell viability analyzer (Vi-CELL, Beckman Coulter Inc.). Clonogenic assays were performed by adding normal growth medium to allow cells to recover following 5 days of drug treatment. Colonies were then stained with crystal violet.

### Transient Transfection

Two million melanoma cells were mixed with 10µg EGFP-LC3 plasmid (ptfLC3) (Addgene) and transfection was carried out using Amaxa Cell Line Nucleofector kit R according to the manufacturer’s instructions (Lonza).

### Lentivirus-mediated Knockdown

pLKO.1-derived vectors with shRNA targeting human *atg7* (TRCN0000007584, TRCN0000007587) or *atg5* (TRCN0000151963 and TRCN0000330394) were purchased from Sigma-Aldrich. Virus was produced using a second-generation packaging system in 293T cells as described [71]. shRNA against Luciferase was generously provided by the Broad Institute and served as a negative control.

### Immunofluorescence

Human Malignant Melanoma Tissue Microarray (US Biomax, Inc) slides, or xenograft tumor paraffin slides were deparaffinized, hydrated and incubated with primary antibody against LC3 (Nanotools, 1∶100 dilution) for 15 min at room temperature followed by FITC tagged anti-mouse (Jackson Immuno Research) antibody, then mounted and visualized by epifluorescence microscopy.

### Three-dimensional Spheroids Growth

3-dimensional collagen matrix cultures were prepared as described [72]. Briefly, a total number of 5000 melanoma cells were suspended in 100µl medium and were overlaid on top of 1.5% agar in each well of 96 well plates. After a 72 hour incubation, the resulting spheroids were collected with a P1000 pipette, transferred to a falcon tube and spun at 2000 rpm for 5 min. By carefully taking away the supernatant, the harvested spheroids were resuspended in diluted collagen matrix. 24 well plates coated with 300 µl of rat tail collagen type I (BD) matrix were prepared and incubated at 37°C until solidified, followed by addition of 1 ml of collagen/spheroids mix to each well. After polymerization, complete medium with or without drug was added on top of the collagen/spheroids matrix and the plates were incubated at 37°C for 72 hours. The cytotoxic effects of the drugs were evaluated by adding calcein-AM and ethidium bromide (LIVE/DEAD Viability/Cytotoxicity Assay Kit, Molecular Probes) according to the manufacturer’s instructions. Pictures of the representative invading spheroids were then taken using epifluorescence microscopy. All conditions were conducted in triplicate.

### Human Melanoma Tumor Xenografts

Animal experiments were performed in accordance with Institutional Animal Care and Use Committee-approved protocols. Six week old male nude mice (NCR Nu-M) were purchased from Taconic and subcutaneously inoculated with melanoma cells UACC903 (1 × 10^6^). When xenografts reached a volume of 100 mm^3^, mice were randomly assigned to 4 different groups (5 mice per group and two xenograft tumors per mouse). Nude mice in these 4 groups received intraperitoneal injection of PBS (vehicle control), HCQ, CCI-779 and the combination of CCI-779 and HCQ, respectively. Tumor growth curves were determined by measuring the tumor size at the indicated time points. Tumors were excised to generate paraffin embedded blocks for immunohistochemistry. Statistical significance was calculated by t-test.

### Immunohistochemistry

Formalin-fixed, paraffin-embedded tissue blocks from either treated or untreated mouse xenograft tumors were deparaffinized and rehydrated by standard procedures. Immunohistochemistry was performed by using an antibody against Ki67 and activated caspase-3 (Cell Signaling).

## Supporting Information

Figure S1 **Knockdown of the essential autophagy gene Atg5 impaired melanoma cell growth.** Western blot shows decreased expression levels of Atg5 (left panel) and impaired clonogenic survival (right panel) in response to lentiviral shRNA knockdown of the essential autophagy regulator Atg5.
(TIF)

Figure S2 **CCI-779 and HCQ-induced melanoma cell death was not rescued by the necroptosis inhibitor Necrostatin 1.** Clonogenic assays were performed by treating melanoma cells with necrostatin 1 at the concentration that blocked renal cell carcinoma cell death [23] for 2 hours before CCI-779 and HCQ were added. No difference in cytotoxicity was observed between cells treated with and without necrostatin 1.
(TIF)
