# Supplementary figures and images for: Coordinate Autophagy and mTOR Pathway Inhibition Enhances Cell Death in Melanoma
Source: PLoS One. 2013 Jan 30;8(1):e55096. doi: 10.1371/journal.pone.0055096 (PMC3559441; doi:10.1371/journal.pone.0055096)

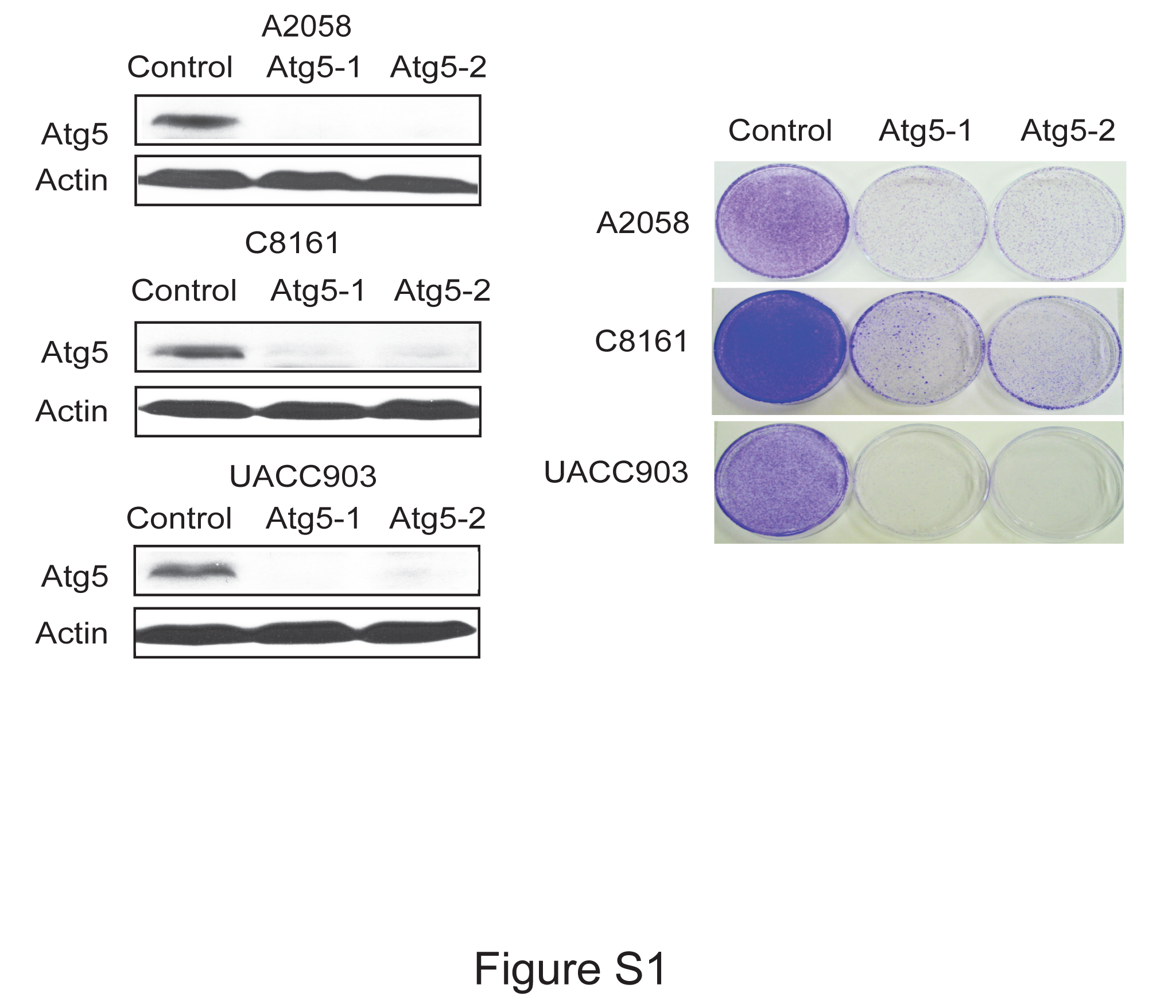

Supplement: Figure S1 — Knockdown of the essential autophagy gene Atg5 impaired melanoma cell growth. Western blot shows decreased expression levels of Atg5 (left panel) and impaired clonogenic survival (right panel) in response to lentiviral shRNA knockdown of the essential autophagy regulator Atg5. (TIF) [file pone.0055096.s001.tif]

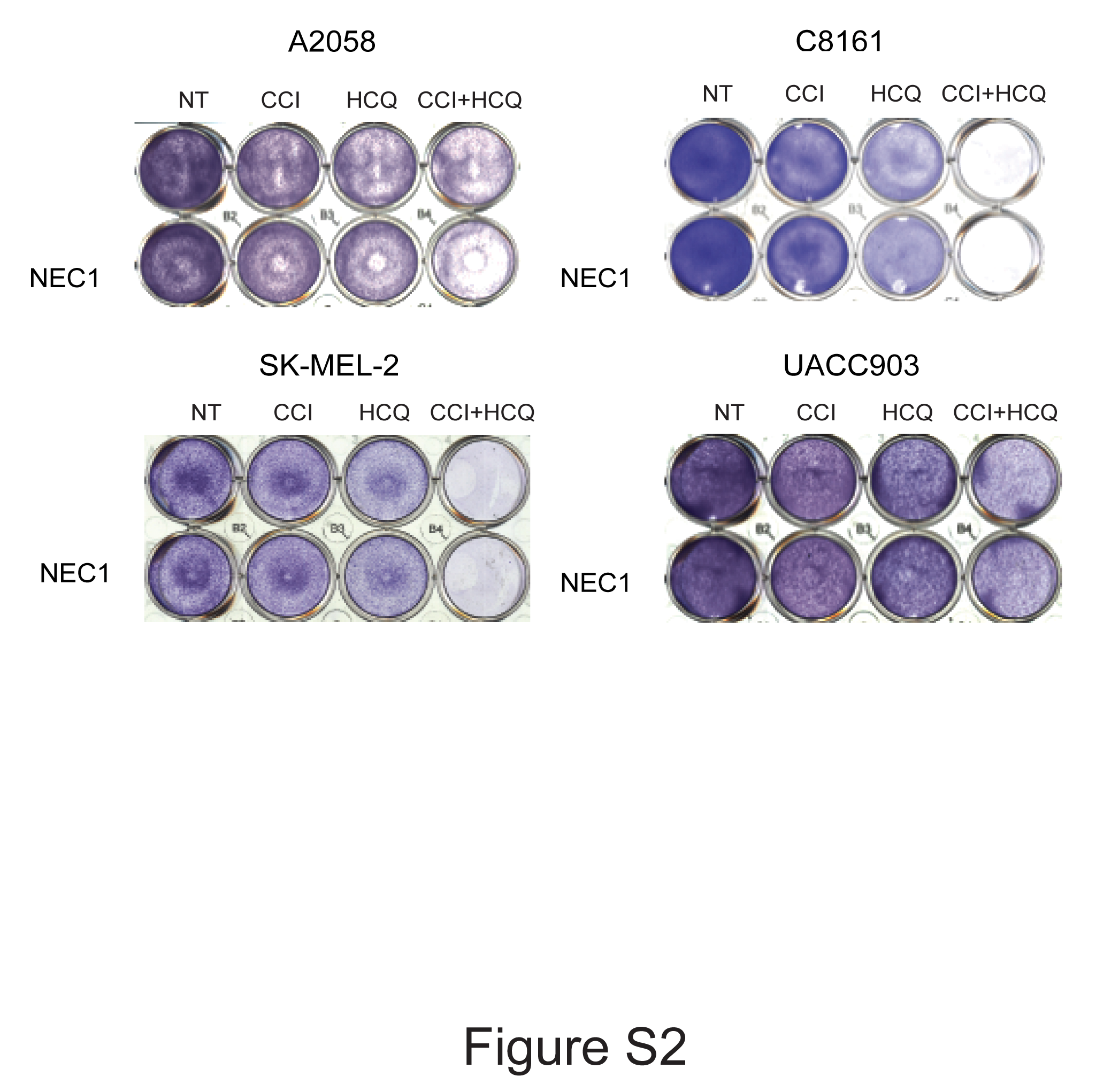

Supplement: Figure S2 — CCI-779 and HCQ-induced melanoma cell death was not rescued by the necroptosis inhibitor Necrostatin 1. Clonogenic assays were performed by treating melanoma cells with necrostatin 1 at the concentration that blocked renal cell carcinoma cell death [23] for 2 hours before CCI-779 and HCQ were added. No difference in cytotoxicity was observed between cells treated with and without necrostatin 1. (TIF) [file pone.0055096.s002.tif]
